# Advanced Glycation Endproducts Are Increased in the Animal Model of Multiple Sclerosis but Cannot Be Reduced by Pyridoxamine Treatment or Glyoxalase 1 Overexpression

**DOI:** 10.3390/ijms19051311

**Published:** 2018-04-27

**Authors:** Suzan Wetzels, Kristiaan Wouters, Toshio Miyata, Jean L. J. M. Scheijen, Jerome J. A. Hendriks, Casper G. Schalkwijk, Tim Vanmierlo

**Affiliations:** 1 Department of Immunology and Biochemistry, Biomedical Research Institute, Hasselt University, Martelarenlaan 42 3500 Hasselt, Belgium; suzan.wetzels@uhasselt.be (S.W.); jerome.hendriks@uhasselt.be (J.J.A.H.); tim.vanmierlo@uhasselt.be (T.V.); 2 Department of Internal Medicine, Cardiovascular Research Institute Maastricht, Maastricht University, 6229 ER Maastricht, The Netherlands; kristiaan.wouters@maastrichtuniversity.nl (K.W.), j.scheijen@maastrichtuniversity.nl (J.L.J.M.S.); 3 Division of Molecular Medicine and Therapy, Tohoku University Graduate School of Medicine, 980-8577 Sendai, Japan; miyata@med.tohoku.ac.jp; 4 Department of Psychiatry & Neuropsychology, School for Mental Health and Neuroscience, Maastricht University, 6229 ER Maastricht, The Netherlands

**Keywords:** advanced glycation endproducts, pyridoxamine, glyoxalase-1, experimental autoimmune encephalomyelitis, multiple sclerosis

## Abstract

Multiple sclerosis (MS) is a demyelinating autoimmune disease of the central nervous system (CNS). The immune response in MS patients leads to the infiltration of immune cells in the CNS and their subsequent activation. Immune cell activation induces a switch towards glycolysis. During glycolysis, the dicarbonyl product methylglyoxal (MGO) is produced. MGO is a glycating agent that can rapidly form advanced glycation endproducts (AGEs). In turn, AGEs are able to induce inflammatory responses. The glyoxalase system is the endogenous defense system of the body to reduce the burden of MGO thereby reducing AGE formation. This system consists of glyoxalase-1 and glyoxalase-2 which are able to detoxify MGO to D-lactate. We investigated whether AGE levels are induced in experimental autoimmune encephalitis (EAE), an inflammatory animal model of MS. Twenty seven days post EAE induction, MGO and AGE (*N*^ε^-(carboxymethyl)lysine (CML), *N*^ε^-(carboxyethyl)lysine (CEL), 5-hydro-5-methylimidazolone (MG-H1)) levels were significantly increased in the spinal cord of mice subjected to EAE. Yet, pyridoxamine treatment and glyoxalase-1 overexpression were unable to counteract AGE production during EAE and did not influence the clinical course of EAE. In conclusion, AGEs levels increase during EAE in the spinal cord, but AGE-modifying treatments do not inhibit EAE-induced AGE production and do not affect disease progression.

## 1. Introduction

Multiple sclerosis (MS) is a demyelinating autoimmune disease of the central nervous system (CNS) [1]. Worldwide, 2.5 million people are diagnosed with MS; mainly young adults as the disease manifests between 20 and 40 years of age. These patients have a variety of symptoms including muscle weakness, paresthesias, ataxia, and visual disturbances, depending on the brain regions attacked by the immune system [2]. Most MS patients, 85%, have the typical relapsing-remitting (RR) MS disease course which results in periods of inflammatory events leading to relapses with clinical disability followed by episodes of full recovery [2]. However, as the disease progresses, over half of the RR MS patients enter a more progressive disease course called secondary progressive (SP) MS, which is characterized by progressive disability without episodes of full recovery [1]. 

The exact etiology of MS remains unknown. However, there is consensus that MS is triggered by environmental factors in genetically susceptible hosts. This leads to an immune response targeted at the myelin sheaths surrounding the axons. Whether this immune response is initiated inside or outside of the CNS is, to date, still unclear. Nevertheless, it is certain that both the innate immune system, comprised of the CNS-resident microglia and the monocyte-infiltrated macrophages, and adaptive immune system, consisting of cell such as the T-lymphocytes and B-lymphocytes play a key role [3]. Autoreactive T-lymphocytes are recruited to the CNS by the secretion of chemokines by infiltrated macrophages and CNS-resident microglia. Once in the CNS, autoreactive T-lymphocytes are reactivated by microglia and infiltrated macrophages thereby promoting neuroinflammation and neurodegeneration [4]. To investigate the development of MS and validate novel therapeutics, animal models of MS have been developed. The experimental autoimmune encephalomyelitis (EAE) is the most widely accepted animal model of MS [5]. Immunization of mice with self-antigens (e.g., myelin oligodendrocyte glycoprotein (MGO)) induces an autoimmune response of CD4^+^ and CD8^+^ T-lymphocytes, B-lymphocytes, and monocytes [6], mimicking the immune response in MS patients. Subsequently, the autoimmune response leads to neuroinflammation and demyelination, primarily in the spinal cord [7].

The activation of CNS-resident microglia and infiltrated macrophages can induce a switch in metabolism in these cells promoting glycolysis over oxidative phosphorylation [8,9]. Increased glycolysis can lead to the formation of the by-product methylglyoxal (MGO) and glyoxal (GO). MGO and GO are potent glycating agents that, reacting with free amino acids, lead to the formation of advanced glycation endproducts (AGEs) [10]. Interaction of MGO with lysine and arginine leads to the formation of *N*^ε^-(1-carboxyethyl)lysine (CEL) and N^δ^-(5-hydro-5-methyl-4-imidazolon-2-yl)-ornithine (MG-H1) respectively, whereas interaction with GO with lysine leads to the formation of *N*^ε^-(carboxymethyl)lysine (CML) [11]. However, whether the pro-inflammatory environment of the CNS in MS leads to the increased formation of AGEs remains to be elucidated. AGEs can contribute to inflammation by binding to their receptor for advanced glycation endproducts (RAGE), resulting in the activation of downstream pathways including nuclear factor-κB (NF-κB), which leads to the production of pro-inflammatory cytokines and oxidative stress [12]. AGEs are known to be increased in diseases in which inflammation is a major component such as atherosclerosis [13,14], obesity [15], non-alcoholic steatohepatitis [16], and diabetes [17]. Moreover, it has been shown that AGEs are present in neurodegenerative diseases such as Alzheimer’s disease and Parkinson’s disease [18,19]. Furthermore, studies have also confirmed the presence of AGEs in the brain and plasma of MS patients [20,21]. This could indicate that during MS, the formation of AGEs may contribute to neuroinflammation, making them a possible therapeutic target. 

To reduce the burden of MGO and AGEs, the body has defense mechanisms such as the glyoxalase system. This system comprises of two enzymes: glyoxalase-1 (Glo-1) and glyoxalase-2 (Glo-2) which are able to detoxify MGO into D-Lactate using glutathione (GSH) as a cofactor [22]. In addition to our body’s defense mechanism, there are also pharmacological agents that are able to lower AGE levels such as pyridoxamine. Pyridoxamine is one of the natural vitamin B6 analogues that scavenges MGO thereby preventing AGE formation [23]. Therefore, both pyridoxamine and Glo-1 stimulating agents are potential therapeutic targets that could be used to reduce AGEs. 

In the current study, we investigated whether AGE levels are induced in the EAE animal model of MS. Moreover, with pyridoxamine treatment and by the Glo-1 overexpression animal model we aimed to reduce AGE levels in the plasma and CNS of mice subjected to EAE, thereby decreasing the disease progression. 

## 2. Results

### 2.1. Experimental Autoimmune Encephalomyelitis Induces the Formation of Advanced Glycation Endproducts in the Central Nervous System

To investigate whether the α-dicarbonyls MGO, GO and 3-deoxyglucosone (3DG) and AGEs CML, CEL and MG-H1 are increased during EAE, we subjected mice to EAE and compared the levels in the plasma, spinal cord and brain with levels of healthy age-matched control mice. MGO, GO and 3DG were significantly decreased in the plasma of mice subjected to EAE (Table 1). Yet, we observed significantly increased levels of free MG-H1 in the plasma of mice subjected to EAE compared to healthy controls (Table 1). Protein bound CML, CEL and MG-H1 were unchanged in the plasma of EAE mice compared to the healthy controls (Table 1).

In the spinal cord, the major site of inflammation during EAE, MGO levels were significantly increased and GO levels tended to be increased (*p* = 0.09) (Table 1). Free CML, CEL and MG-H1, but not protein bound CML, CEL and MG-H1, were significantly increased in the spinal cord of mice subjected to EAE (Table 1). To determine whether the main MGO detoxification enzyme is affected during EAE, Glo-1 activity was determined in the spinal cord. Glo-1 activity was significantly reduced in the spinal cord of mice subjected to EAE compared to their healthy wild type (WT) controls (Table 1). Glo-2 activity was equal between the groups [24]. 

MGO and GO levels were also significantly increased in the brain of mice subjected to EAE (Table 1). In addition, free CML was increased in the brain whereas free CEL and MG-H1 remained unchanged. Protein bound CML, CEL and MG-H1 was also not altered in the brain of mice subjected to EAE compared to healthy controls (Table 1). Glo-1 activity in the brain however was increased in the EAE mice (Table 1). Glo-2 activity between the two groups was not significantly altered due to EAE [24].

### 2.2. Pyridoxamine Intervention did not Reduce Advanced Glycation Endproduct Levels in the Plasma and Spinal Cord During EAE

**Figure 1:**
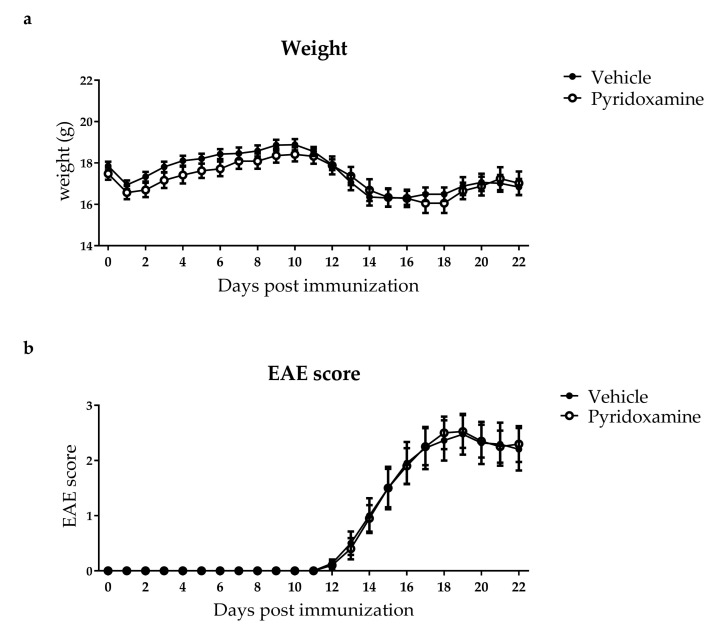
10 g/L Pyridoxamine does not affect EAE disease outcome. Mice were immunized with myelin oligodendrocyte glycoprotein (MOG) to induce EAE and treated with either vehicle or 10 g/L pyridoxamine. Vehicle (*n* = 11) and 10 g/L pyridoxamine (*n* = 10) treated mice were weighed (**a**) and scored (**b**) daily for 22 days after EAE induction. Closed circles (●) represent vehicle treated mice and open circles (○) represent 10 g/L pyridoxamine treated mice. Data is presented as mean ± SEM and analyzed using two-way analysis of variance (ANOVA) with Sidak’s multiple comparisons post-test.

To investigate whether inhibition of AGE formation attenuates disease progression in an animal model of MS, mice were treated with 10 g/L pyridoxamine via oral gavage during EAE. Pyridoxamine is described as a dicarbonyl scavenger, thereby reducing AGE levels [23]. The control group was treated with vehicle. Analysis of plasma and spinal cord of mice treated with pyridoxamine and vehicle showed that dicarbonyls, free AGEs and protein bound AGEs were not altered in the pyridoxamine treated group compared to the vehicle treated group after EAE (Table 2). Glo-1 activity in the spinal cord was not altered between the groups (Table 2). In line, pyridoxamine treatment did no lead to differences in weight, and disease progression, measured by EAE score, compared to vehicle treated controls (Figure 1A,B).

### 2.3. Human Glo-1 Overexpression Mouse Model did not Reduce Advanced Glycation Endproduct Levels in the Plasma and Central Nervous System during EAE

10 g/L Pyridoxamine treatment was unable to reduce dicarbonyl and AGE levels in the plasma and central nervous system. As an alternative mouse model to lower AGEs during EAE development, the Glo-1 overexpression mouse model was used. In this mouse model, the human Glo-1 gene is inserted under the control of the beta-actin promoter, leading to a full body overexpression of the Glo-1 enzyme. Wild-type litter mates were used as a control group. EAE was induced in both Glo-1 overexpression mice and wild type mice.

**Figure 2:**
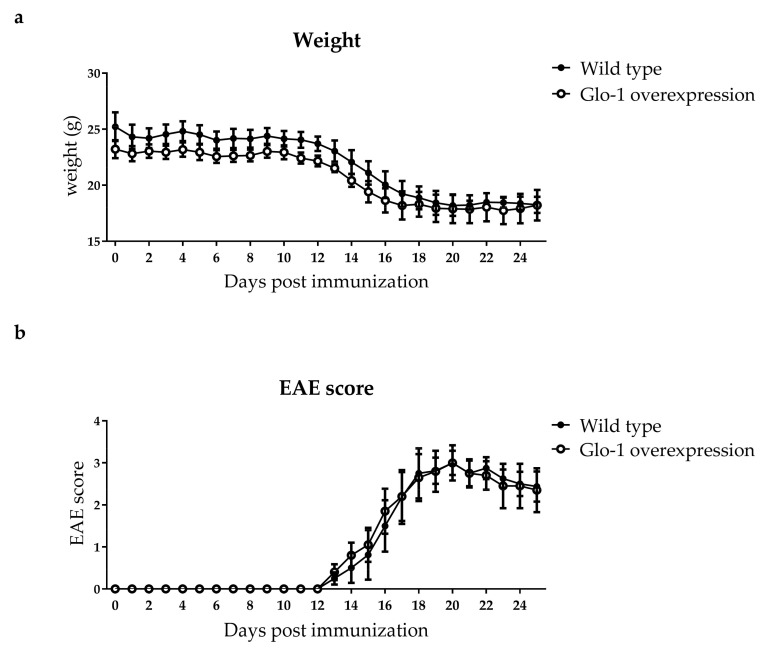
Full body Glo-1 overexpression does not affect EAE disease outcome. Glo-1 overexpression mice and wild type littermates were immunized with MOG to induce EAE. Wild type (*n* = 4) and Glo-1 overexpression (*n* = 5) mice were weighed (**a**) and scored (**b**) daily for 25 days after EAE induction. Closed circles (●) represent wild type littermates and open circles (○) represent Glo-1 overexpression mice. Data is presented as mean ± SEM and analyzed using two-way ANOVA with Sidak’s multiple comparisons post-test.

Glo-1 activity was assessed in the CNS of the Glo-1 overexpression and wild type mice after EAE. Glo-1 activity was 5.5 and 5.9 times increased in respectively the spinal cord and brain of Glo-1 overexpression mice compared to their wild type littermates (Table 3). However, dicarbonyl levels and free and protein-bound AGE levels in the plasma, spinal cord, and brain were similar after EAE (Table 3). In line with this, weight reduction and clinical EAE score were not altered in the Glo-1 overexpression mice compared to the wild type controls (Figure 2A,B).

## 3. Discussion

Our study shows that levels of dicarbonyls and AGEs are significantly increased in the CNS of mice subjected to EAE. Moreover, Glo-1 activity in the spinal cord was significantly decreased whereas Glo-1 is increased in the brain. Therapeutic approaches to reduce AGE levels using pyridoxamine and human Glo-1 overexpression failed to reduce AGE levels in the plasma and CNS of EAE mice, which was paralleled with unchanged neurological scores.

EAE progression leads to a decrease of plasma levels of MGO, GO and 3DG but an increase of free MG-H1 compared to healthy controls. The decrease in dicarbonyl levels in the plasma could indicate that plasma MGO, GO and 3DG are able to pass the blood-brain barrier and accumulate in the spinal cord. Here, dicarbonyls are able to induce the formation of free CML, CEL and MG-H1 since GO and 3DG lead to the formation of CML, whereas MGO leads to the formation of CEL and MG-H1. We have found increased levels of CML, CEL and MG-H1 in the spinal cord of mice subjected to EAE. This suggests that plasma dicarbonyls enter the CNS and leads to a decrease in the plasma and increased formation of AGEs in the CNS. Moreover, MGO, GO, free CML, CEL and MG-H1 are increased in the spinal cord of EAE mice compared to healthy animals. We also observed decreased Glo-1 activity levels in the spinal cord. The increase of AGEs in the spinal cord may be due to the combination of infiltration and activation of immune cells and decreased Glo-1 activity. The activation of microglia and infiltrated macrophages leads to an induction of glycolysis [8,9], probably resulting in the formation of MGO and GO. Moreover, Bogie et al. showed that phagocytosis of myelin by macrophages induces genes involved in glycolysis [25], also potentially contributing to local MGO production. In addition, Hanssen et al. revealed that inflammatory cytokine tumor necrosis factor (TNF) reduces Glo-1 activity in U937 monocytes in vitro [13], suggesting that the pro-inflammatory environment in the spinal cord of EAE mice contributes to the decrease in Glo-1 activity and the concomitant increase in AGE levels. 

In contrast to the spinal cord, in the brain only MGO, GO and CML were significantly increased and Glo-1 activity was even increased compared to healthy controls. Since Glo-1 detoxifies MGO and prevents the formation of MGO-derived AGEs such as CEL and MG-H1, this explains why CEL and MG-H1 were not increased in the brain of EAE mice. In addition, inflammatory demyelinating lesions are more abundant in the spinal cord compared to the brain of the EAE model [26]. It is therefore conceivable that inflammation-induced AGE production is of more importance in the spinal cord compared to the brain. 

AGEs are known to bind to their receptor RAGE resulting in NF-κB activation and subsequent production of pro-inflammatory cytokines [12]. Lowering of AGE levels in the spinal cord during EAE could therefore reduce inflammation and neurological disease progression. Indeed, Yan et al. have shown that prevention of RAGE activation by soluble RAGE (sRAGE) and inhibition of RAGE activation on CD4^+^ T-cells leads to a decreased EAE disease progression [27]. These results suggest that lowering RAGE ligands could ameliorate neuroinflammatory responses.

One potential AGE lowering substance is pyridoxamine. Pyridoxamine is one of the three natural vitamin B6 vitamers along with pyridoxine and pyridoxal. Pyridoxamine is described to lower AGEs by scavenging dicarbonyls such as MGO [28]. Food-derived pyridoxamine is absorbed in the intestine by means of passive diffusion [29]. After uptake of pyridoxamine, pyridoxamine is converted into pyridoxamine-5’-phosphate and further into pyridoxal-5’-phosphate in the intestine and liver [30,31]. Sakurai et al. found that supplementation with higher concentrations of labelled [3H]pyridoxamine (140 nmol) resulted in a significant amount of labelled pyridoxamine, pyridoxal and pyridoxal-5’-phosphate in the plasma [30,31]. In our current study, we have administered 0.5 mL of 10 g/L pyridoxamine via oral gavage twice daily which results in a concentration of 59.5 mmol daily which is significantly higher compared to Sakurai et al. Moreover, van der Ham et al. developed a UPLC MSMS (Ultra-performance liquid chromatography tandem mass spectrometry) method for the quantification of the vitamin B6 vitamers pyridoxamine, pyridoxine, and pyridoxal and has proven that pyridoxine supplementation increases the levels of all three vitamers including pyridoxamine in the cerebrospinal fluid of these persons [32], indicating that the free form of pyridoxamine, and also pyridoxine and pyridoxal, are able to cross the blood-brain barrier thereby entering the cerebrospinal fluid (CSF). Subsequently, brain cells are capable of active uptake of pyridoxamine and the other two forms from the CSF [33]. Pyridoxamine supplementation for 24 weeks has been proven to reduce AGE levels in clinical trials studying osteoarthritis and diabetic nephropathy [34,35]. Moreover, we have previously shown that pyridoxamine treatment inhibits adipose tissue expansion and -induced adipose tissue inflammation, indicating that pyridoxamine is capable to reduce inflammation in vivo using a 5 times lower dose [36]. However, in this current study, we have found that pyridoxamine was not capable to affect AGE levels in the plasma and spinal cord during EAE, which may be due to the severity of the animal model of MS which possibly limits major decreases in AGE formation. 

We found that Glo-1 activity is significantly reduced in the spinal cord of mice subjected to EAE compared to healthy controls. Since Glo-1 is the major enzyme involved in the detoxification of MGO and thereby preventing the formation of AGEs, we have used Glo-1 overexpression mice. The inserted human Glo-1 is under the control of the β-actin promoter [37], and in the CNS, microglia are the main cells with high β-actin transcription [38], suggesting that microglia have high overexpression of Glo-1 resulting in a high capacity to detoxify MGO and prevent AGE formation. Although we confirmed that Glo-1 overexpressing mice have approximately 5.5–5.9 times higher Glo-1 activity in brain and spinal cord compared to their wild type littermates, we observed equal dicarbonyl and AGE levels in Glo-1 overexpressing and wild type littermates. The detoxification of MGO via the glyoxalase pathway requires GSH as the initial step of the pathway [39]. It has previously been shown that GSH levels are reduced in the acute phase of EAE [40,41]. It is therefore possible that, due to decreased availability of GSH in the spinal cord of mice subjected to EAE, Glo-1 overexpression may not result in increased Glo-1 activity in vivo.

In conclusion, we have revealed that dicarbonyl and AGE levels are increased in the experimental animal model of MS. This may suggest that similar pathways are activated in MS patients, as recently reviewed [42]. However, we were unable to reduce AGE levels by pyridoxamine treatment and a Glo-1 overexpression in the EAE model. This model is an acute model which mimics the initial response in MS patients. Nevertheless, MS is a chronic disease with several disease phases and underlying disease pathologies which are not all simulated in the acute EAE model. Therefore, we cannot exclude the possibility that AGE lowering therapies could be beneficial for MS patients.

## 4. Materials and Methods

### 4.1. Animal Experiments

First, sixteen 9-week old female C57Bl/6JOlaHsd mice were purchased (Envigo, Venray, The Netherlands) and randomly distributed over two groups (*n* = 8). Mice were left to acclimatize for 9 days in the animal facility. Experimental autoimmune encephalomyelitis (EAE) was induced according to manufacturer’s instructions (Hooke Laboratories, St. Lawrence, MA, USA) using 200 µg myelin oligodendrocyte glycoprotein (MOG_35–55_) emulsified in 200 µL complete freund’s adjuvant (CFA) containing 5 mg/mL Mycobacterium tuberculosis and 100 ng pertussis toxin. Mice were weighed and scored for EAE disease progression using a scale for neurological symptoms: 0 = no neurological symptoms, 0.5 = limp tail tip, 1 = complete limp tail, 1.5 = limp tail and hind leg inhibition, 2 = limp tail and weakness of both hind limbs, 2.5 = limp tail and dragging of hind limbs, 3 = limp tail and complete paralysis of hind limbs, 3.5 = limp tail, complete paralysis of hind limbs and mouse is unable to right itself when placed on the side, 4 = limb tail, complete hind limb and partial front limb paralysis, mouse remains alert, 4.5 = limb tail, complete hind limb and partial front limb paralysis, mouse is not alert, 5 = moribund or death due to EAE. After 27 days of EAE, mice were euthanized with Nembutal (Val d’Hony-Verdifarm, Beringen, Belgium) and plasma, spinal cord and brain was isolated for further analysis.

Second, twenty-two 10-week old female C57Bl/6JOlaHsd mice were purchased (Envigo, Venray, The Netherlands) and randomly distributed over two cages (*n* = 11). Mice were able to acclimatize for 7 days in the animal facility before the start of the intervention. To reduce AGE formation, the vitamin B6 analogue pyridoxamine (10 g/L) was administered by 0.5 mL oral gavage twice daily. Pyridoxamine was kindly provided by Prof. Miyata and functionality was tested in an in vitro setup. To prevent any cage-effects, mice were randomly assigned to the intervention or control group in both cages. Administration of vehicle or intervention was done blinded. After two days of intervention, EAE was induced as described above. Mice continued with the twice daily intervention of pyridoxamine or vehicle during EAE and were weighed and scored as described above. Mice received the last dose of pyridoxamine via oral gavage 22 days after induction of EAE. Two hours after oral gavage, mice were euthanized with Nembutal (Val d’Hony-Verdifarm, Beringen, Belgium) and plasma and spinal cord was isolated for further analysis.

Finally, heterozygous C57Bl/6J mice with a universal overexpression of the human Glo-1 enzyme were kindly provided by Prof. Miyata [37]. Female heterozygous Glo-1 overexpressing mice (*n* = 5) and wild type littermates (*n* = 5) were used. At 11–13 weeks of age, EAE was induced as described above. Mice were weighed and scored daily. After 25 days of EAE, mice were euthanized with Nembutal (Val d’Hony-Verdifarm, Beringen, Belgium) and plasma, spinal cord and brain was isolated for further analysis. 

All experiments were approved by the local ethical committee for animal experiments of Hasselt University and performed according to the institutional guidelines (201557 approved on 8-01-2016, 201557A1 approved on 29-04-2016, 201636 approved on 25-09-2016).

### 4.2. α-Dicarbonyl and AGE Measurements

Spinal cord and brain were used to make 5% protein homogenates in 0,1 M sodium phosphate buffer (pH 6.8) supplemented with protease inhibitor (Roche, Basel, Switzerland) and 0.02% Triton-x.

The dicarbonyls methylglyoxal (MGO), glyoxal (GO) and 3-deoxyglucosone (3DG), and the free and protein-bound form of AGEs *N*^ε^-(carboxymethyl)lysine (CML), *N*^ε^-(1-carboxyethyl)lysine (CEL), and *N*^δ^-(5-hydro-5-methyl-4-imidazolon-2-yl)-ornithine (MG-H1) were analysed in the plasma, spinal cord and brain of mice using ultra-performance liquid chromatography tandem mass spectrometry (UPLC MSMS) as described previously [13,43].

### 4.3. Glyoxalase-1 Activity Assay

Glyoxalase-1 (Glo-1) activity was measured in protein homogenates of the spinal cord and brain as previously described by McLellan et al. [44]. In short, Glo-1 activity was determined by measuring the formation of S-d-Lactoylglutathione from MGO at an absorbance of 240 nm during 30 min using a spectrophotometry analysis.

### 4.4. Glyoxalase-2 Activity Assay

Glyoxalase-2 (Glo-2) activity was measured in protein homogenates of the spinal cord and brain as previously described by Arai et al. [45]. The activity assay mix consisted of 0.3 mM S-d-Lactoylglutathione (Sigma-Aldrich, Saint Louis, MO, USA) diluted in a 50 mM Tris-HCl buffer (pH 7.4). Using a spectrophotometry analysis, Glo-2 activity was determined as the degradation of S-d-Lactoylglutathione measured at an absorbance of 240 nm during 30 min.

### 4.5. Statistical Analysis

Data is presented as mean ± standard error of the mean (SEM). Statistical analysis was performed with GraphPad Prism version 7 (GraphPad Software, La Jolla, CA, USA). Data is analyzed using unpaired t-test and two-way ANOVA with Sidak’s multiple comparisons post-test. A *p* ≤ 0.05 was considered statistically significant.

## Figures and Tables

**Table 1:** Dicarbonyls and free advanced glycation endproducts (AGEs) in the plasma, spinal cord, and brain of mice subjected to experimental autoimmune encephalitis (EAE) and healthy controls.

Substrate	Product	Healthy *n* = 8	EAE *n* = 8	*p*-Value
Plasma				
	Methylglyoxal (MGO) (nmol/L)	3268 ± 378	2160 ± 230	0.03
	Glyoxal (GO) (nmol/L)	1414 ± 51	1205 ± 70	0.03
	3-deoxyglucosone (3DG) (nmol/L)	2051 ± 123	1611 ± 152	0.04
	Protein bound *N*^ε^-(carboxymethyl)lysine (CML) (nmol/mmol Lysine)	25.6 ± 1	25.2 ± 1	0.83
	Protein bound *N*^ε^-(1-carboxyethyl)lysine (CEL) (nmol/mmol Lysine)	9.1 ± 0.7	8.4 ± 1	0.60
	Protein bound N^δ^-(5-hydro-5-methyl-4-imidazolon-2-yl)-ornithine (MG-H1) (nmol/mmol Lysine)	276.5 ± 19	285.6 ± 17	0.73
	Free CML (nmol/L)	315.4 ± 17	283.6 ± 15	0.18
	Free CEL (nmol/L)	98.1 ± 7	99.9 ± 9	0.88
	Free MG-H1 (nmol/L)	59.6 ± 6	91.5 ± 6	0.002
Spinal cord				
	MGO (nmol/L)	1980 ± 169	3143 ± 419	0.02
	GO (nmol/L)	1706 ± 250	2258 ± 165	0.09
	Protein bound CML (nmol/mmol Lysine)	297.5 ± 116	187.2 ± 16	0.36
	Protein bound CEL (nmol/mmol Lysine)	65.1 ± 16	37.4 ± 2	0.11
	Protein bound MG-H1 (nmol/mmol Lysine)	159.4 ± 10	165.3 ± 14	0.73
	Free CML (nmol/L)	61 ± 2	98.18 ± 8	0.0005
	Free CEL (nmol/L)	14.6 ± 0.4	18.1 ± 1	0.01
	Free MG-H1 (nmol/L)	3.4 ± 0.07	5.5 ± 0.8	0.01
	Glo-1 activity (nmol/mg/min)	262.5 ± 9	198.9 ± 14	0.002
Brain				
	MGO (nmol/L)	1222 ± 162	2302 ± 299	0.01
	GO (nmol/L)	1833 ± 169	2250 ± 89	0.05
	Protein bound CML (nmol/mmol Lysine)	91.5 ± 5	98.7 ± 6	0.36
	Protein bound CEL (nmol/mmol Lysine)	42.6 ± 2	43.7 ± 3	0.77
	Protein bound MG-H1 (nmol/mmol Lysine)	129.5 ± 16	139.4 ± 19	0.70
	Free CML (nmol/L)	55.6 ± 2	73.9 ± 3	<0.0001
	Free CEL (nmol/L)	24.6 ± 0.8	25.3 ± 1	0.69
	Free MG-H1 (nmol/L)	3.5 ± 0.2	4.0 ± 0.3	0.19
	Glo-1 activity (nmol/mg/min)	147.9 ± 5	161.4 ± 3	0.04

Data presented as mean ± standard error of mean (SEM) and analyzed using unpaired *t*-test.

**Table 2:** Dicarbonyl and AGE levels in the plasma and spinal cord after daily oral vehicle or pyridoxamine (10 g/L) treatment during EAE.

Substrate	Product	Vehicle *n* = 11	Pyridoxamine *n* = 10	*p*-Value
Plasma				
	MGO (nmol/L)	2270 ± 208	2488 ± 227	0.49
	GO (nmol/L)	1447 ± 159	1391 ± 91	0.77
	3DG (nmol/L)	1496 ± 89	1642 ± 44	0.17
	Protein bound CML (nmol/mmol Lysine)	19.7 ± 1	20 ± 2	0.89
	Protein bound CEL (nmol/mmol Lysine)	5.3 ± 0.4	4.7 ± 0.4	0.31
	Protein bound MG-H1 (nmol/mmol Lysine)	217.0 ± 12	239.2 ± 15	0.25
	Free CML (nmol/L)	364.5 ± 137	218.4 ± 10	0.32
	Free CEL (nmol/L)	138.8 ± 70	71.6 ± 6	0.37
	Free MG-H1 (nmol/L)	96.6 ± 37	63.6 ± 7	0.42
Spinal cord				
	MGO (nmol/L)	1976 ± 177	1977 ± 132	1.00
	GO (nmol/L)	2117 ± 122	2207 ± 150	0.65
	Protein bound CML (nmol/mmol Lysine)	97.6 ± 12	95.9 ± 12	0.92
	Protein bound CEL (nmol/mmol Lysine)	50.2 ± 4	52.2 ± 5	0.75
	Protein bound MG-H1 (nmol/mmol Lysine)	108.3 ± 11	132.1 ± 17	0.25
	Free CML (nmol/L)	92.9 ± 5	97.3 ± 5	0.56
	Free CEL (nmol/L)	17.6 ± 0.4	17.9 ± 0.5	0.59
	Free MG-H1 (nmol/L)	4.5 ± 0.2	4.8 ± 0.3	0.43
	Glo-1 activity (nmol/mg/min)	1614 ± 46	1572 ± 59	0.58

Data presented as mean ± SEM and analyzed using unpaired *t*-test.

**Table 3:** Dicarbonyl and AGE levels in the plasma and central nervous system of full body Glo-1 overexpression mice and wild type littermates after EAE.

Substrate	Product	Wild Type *n* = 4	Glo-1 Overexpression *n* = 5	*p*-Value
Plasma				
	MGO (nmol/L)	1837 ± 578	3279 ± 432	0.08
	GO (nmol/L)	1047 ± 44	1477 ± 339	0.30
	3DG (nmol/L)	1690 ± 172	2125 ± 167	0.11
	Protein bound CML (nmol/mmol Lysine)	25.9 ± 0. 8	26.9 ± 2	0.64
	Protein bound CEL (nmol/mmol Lysine)	6.2 ± 1	8.8 ± 0.8	0.08
	Protein bound MG-H1 (nmol/mmol Lysine)	307.9 ± 51	274.0 ± 15	0.50
	Free CML (nmol/L)	251.1 ± 28	790.4 ± 523	0.39
	Free CEL (nmol/L)	72.7 ± 15	330.4 ± 250	0.39
	Free MG-H1 (nmol/L)	64.0 ± 8	169.1 ± 114	0.44
Spinal cord				
	MGO (nmol/L)	3865 ± 500	3382 ± 184	0.35
	GO (nmol/L)	2794 ± 630	2517 ± 220	0.66
	Protein bound CML (nmol/mmol Lysine)	108.4 ± 4	102.3 ± 8	0.54
	Protein bound CEL (nmol/mmol Lysine)	41.9 ± 6	46.5 ± 9	0.69
	Protein bound MG-H1 (nmol/mmol Lysine)	270.7 ± 25	305.4 ± 47	0.57
	Free CML (nmol/L)	87.3 ± 9	92.9 ± 7	0.64
	Free CEL (nmol/L)	17.1 ± 0.8	16.4 ± 0.5	0.53
	Free MG-H1 (nmol/L)	4.4 ± 0. 8	3.7 ± 0.1	0.32
	Glo-1 activity (nmol/mg/min)	66.6 ± 8	367.8 ± 6	<0.0001
Brain				
	MGO (nmol/L)	2906 ± 309	2836 ± 127	0.83
	GO (nmol/L)	4086 ± 718	4291 ± 354	0.79
	Protein bound CML (nmol/mmol Lysine)	63.9 ± 4	57.8 ± 7	0.50
	Protein bound CEL (nmol/mmol Lysine)	40.6 ± 6	37.0 ± 2	0.55
	Protein bound MG-H1 (nmol/mmol Lysine)	177.3 ± 16	179.4 ± 13	0.92
	Free CML (nmol/L)	52.0 ± 5	51.2 ± 3	0.88
	Free CEL (nmol/L)	25.4 ± 2	25.4 ± 1	0.97
	Free MG-H1 (nmol/L)	3.3 ± 0.1	3.4 ± 0.2	0.69
	Glo-1 activity (nmol/mg/min)	50.1 ± 2	296.1 ± 11	<0.0001

Data presented as mean ± SEM and analyzed using unpaired *t*-test.

## Abbreviations

3DG	3-deoxyglucosone
AGEs	Advanced glycation endproducts
CEL	*N*^ε^-(1-carboxyethyl)lysine
CFA	Complete freund’s adjuvant
CML	*N*^ε^-(carboxymethyl)lysine
CNS	Central nervous system
CSF	Cerebrospinal fluid
EAE	Experimental autoimmune encephalomyelitis
Glo-1	Glyoxalase-1
Glo-2	Glyoxalase-2
GO	Glyoxal
GSH	Glutathione
MG-H1	*N*^δ^-(5-hydro-5-methyl-4-imidazolon-2-yl)-ornithine
MGO	Methylglyoxal
MOG	Myelin oligodendrocyte glycoprotein
MS	Multiple sclerosis
NF-κB	Nuclear factor-κB
RAGE	Receptor for advanced glycation endproducts
RR MS	Relapsing-remitting MS
SP MS	Secondary progressive MS
sRAGE	Soluble receptor for advanced glycation endproducts
UPLC MSMS	Ultra-performance liquid chromatography tandem mass spectrometry
